# Artesunate overcomes drug resistance in multiple myeloma by inducing mitochondrial stress and non-caspase apoptosis

**DOI:** 10.18632/oncotarget.1847

**Published:** 2014-03-24

**Authors:** Xenofon Papanikolaou, Sarah Johnson, Tarun Garg, Erming Tian, Ruslana Tytarenko, Qing Zhang, Caleb Stein, Bart Barlogie, Joshua Epstein, Christoph Heuck

**Affiliations:** ^1^ Myeloma Institute for Research and Therapy, University of Arkansas for Medical Sciences, Little Rock, AR

**Keywords:** artesunate, apoptosis, caspases, myeloma, ROS

## Abstract

Although novel drugs have contributed immensely to improving outcomes of patients with multiple myeloma (MM), many patients develop drug resistance and ultimately succumb to MM. Here, we show that artesunate, an anti-malarial drug, reliably induces cell death in vitro in naïve as well as drug-resistant MM cells at concentrations shown to be safe in humans. Artesunate induced apoptosis predominantly through the non-caspase mediated pathway by primarily targeting mitochondria and causing outer mitochondrial membrane permeabilization that led to cytosolic and subsequent nuclear translocation of mitochondrial proteins apoptosis inducing factor (AIF) and endonuclease G (EndoG). Nuclear translocation of AIF and EndoG was accompanied by low levels of reactive oxygen species (ROS) and increased mitochondrial production of superoxide. These effects were present before apoptosis was evident and were related to intracellular levels of bivalent iron (Fe^+2^). Artesunate's unique mechanism probably was at least partially responsible for, its ability to act synergistically with multiple anti-myeloma agents. Our findings suggest that artesunate acts through iron to affect the mitochondria and induce low ROS and non-caspase–mediated apoptosis. Its potency, toxicity profile, and synergism with other drugs make it an intriguing new candidate for MM treatment.

## INTRODUCTION

With therapeutic advances over the last two decades, 5-year life expectancy for patients diagnosed with multiple myeloma (MM) has increased from 15% [1] to 70% [2]. Nevertheless, treatment of patients diagnosed with high-risk MM [3] or those who relapse after exposure to a multitude of anti-MM agents remains a significant challenge. For instance, Kumar et al. recently showed that MM patients who developed resistance to bortezomib (BZ) and IMiDs have an ominous median survival prognosis of only 9 months [4]. This patient population is in clear need of new drugs without cross-resistance to existing agents and, ideally, that function through a mechanism that has not yet been exploited for therapy.

Recent research has demonstrated that artemisin-based endoperoxide drugs can function as experimental cancer chemotherapeutics with significant activity in cell-based and animal studies [5-8]. These drugs constitute an important class of FDA-approved antimalarial agents, of which artesunate (ART) is the most potent available today [9]. ART's clinically proven favorable toxicity profile for malaria treatment [9], combined with its potential as a cancer chemotherapeutic, prompted us to examine its *in vitro* anti-MM activity.

In this study we establish the *in-vitro* effectiveness of ART against MM and in MM models of BZ resistance. Furthermore, we show that ART induces apoptosis mainly through the non-caspase mediated pathway, primarily targeting mitochondria, and that the effect is related to intracellular levels of bivalent iron. MM cells' “commitment” to apoptosis after treatment with ART is characterized by low cellular levels of reactive oxygen species (ROS). These unconventional mechanisms of action could help explain the absence of cross-resistance to ART in MM models of *in-vitro* established chemoresistance and the synergism between ART and different frontline anti-myeloma agents.

## RESULTS

### ART inhibits viability of MM cell lines and primary MM cells, regardless of prior drug resistance, in a dose- and time-dependent manner

**Figure 1:**
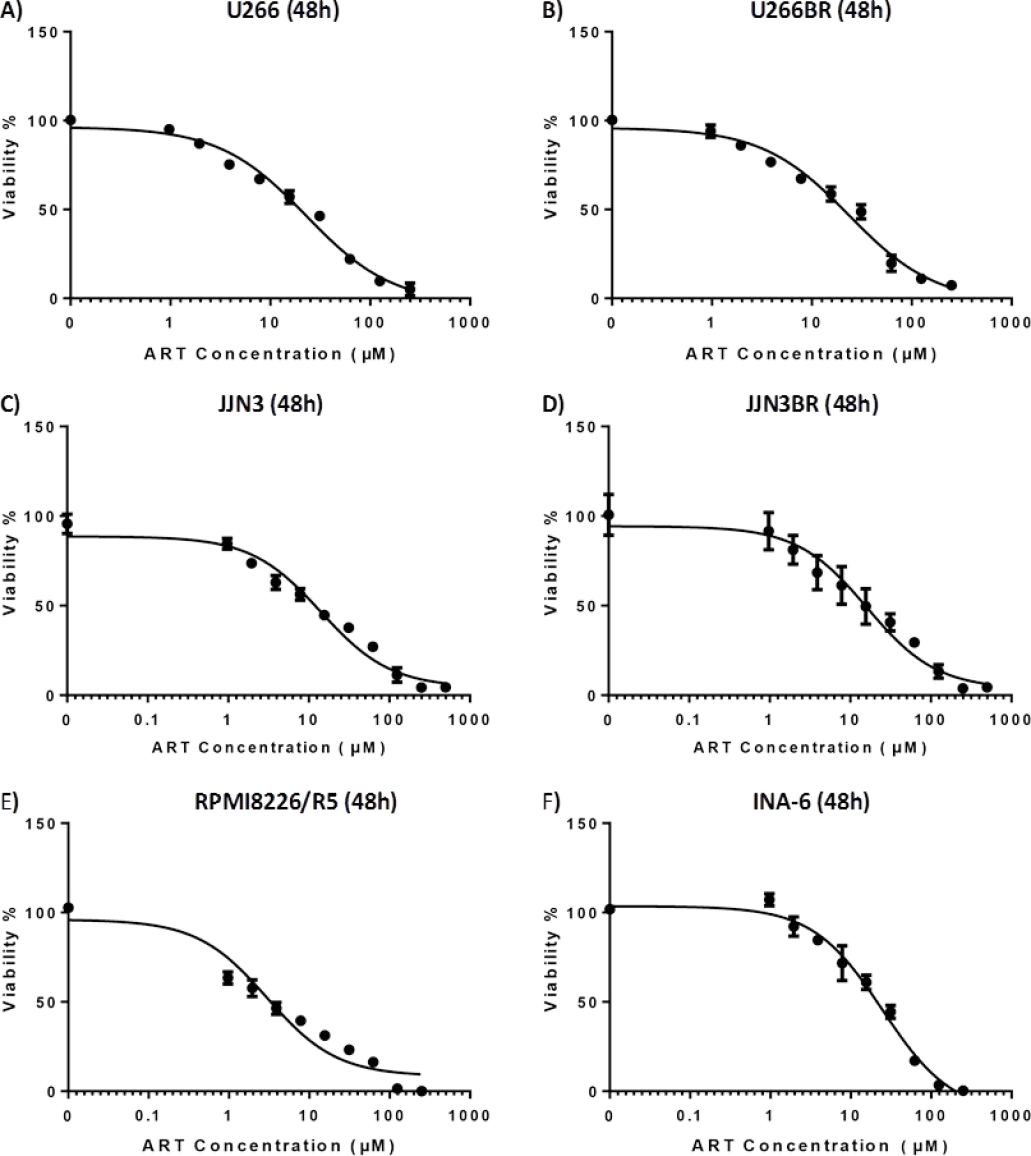
IC50 curves of various cell lines on 48 hour exposure to ART A) U266 cell line B)U266BR cell line C) JJN3 cell line D) JJN3BR cell line E) RPMI8226/R5 F)INA-6 cell line

To assess the efficacy of ART as an antimyeloma agent, we first evaluated its impact on the viability of the MM cells. ART was able to inhibit viability in clinically achievable concentrations [10] and in a time-dependent manner in IL-6-independent and -dependent MM cell lines (Suppl. Table S1A; Fig. 1A-E). The MM cell line RPMI 8226/R5 which is known to exhibit one of the highest BZ IC50s among MM cell lines [11] proved to be among the most sensitive to ART.

Motivated by this finding and by the clinical importance of drug resistance in treating MM, we sought to investigate the effects of ART in different models of drug resistance. We developed two BZ-resistant (BR) sublines of BZ-sensitive cell lines JJN3 and U266 by exposing cells to increasing concentrations of BZ. JJN3BR and U266BR exhibited 20-fold increases in BZ IC50 (48 h), compared to BZ-naïve parental cells (Supplementary Fig. S1A and B). Treating JJN3BR and U266BR cells with ART resulted in no evidence of cross-resistance, compared to parental cell lines; similarly treating dexamethasone (DEX)-resistant cell line MM-1R and its DEX-sensitive mate MM.1S resulted also in no cross-resistance. *In-vitro* anti-MM effectiveness of ART was also evident in CD138-selected primary MM cells from six patients with relapsed/refractory MM (Supplementary Table S1B).

### ART induces non-caspase mediated apoptosis in MM cell lines

**Figure 2:**
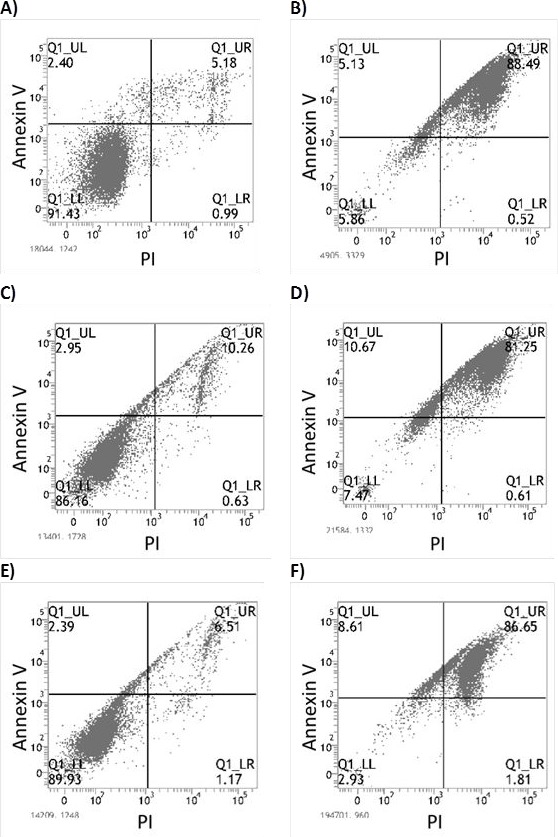
Flow cytometry assay for detection of early and late apoptosis through Annexin V and PI staining respectively A) Untreated JJN3 cells (Control) B) JJN3 after 48 hour exposure to 125μM ART C) Untreated JJN3-BR cells (Control) D) JJN3-BR after 48 hour exposure to 125μM ART E) Untreated RPMI-8226 cells (Control) F) RPMI-8226 after 48 hour exposure to 125μM ART

**Figure 3:**
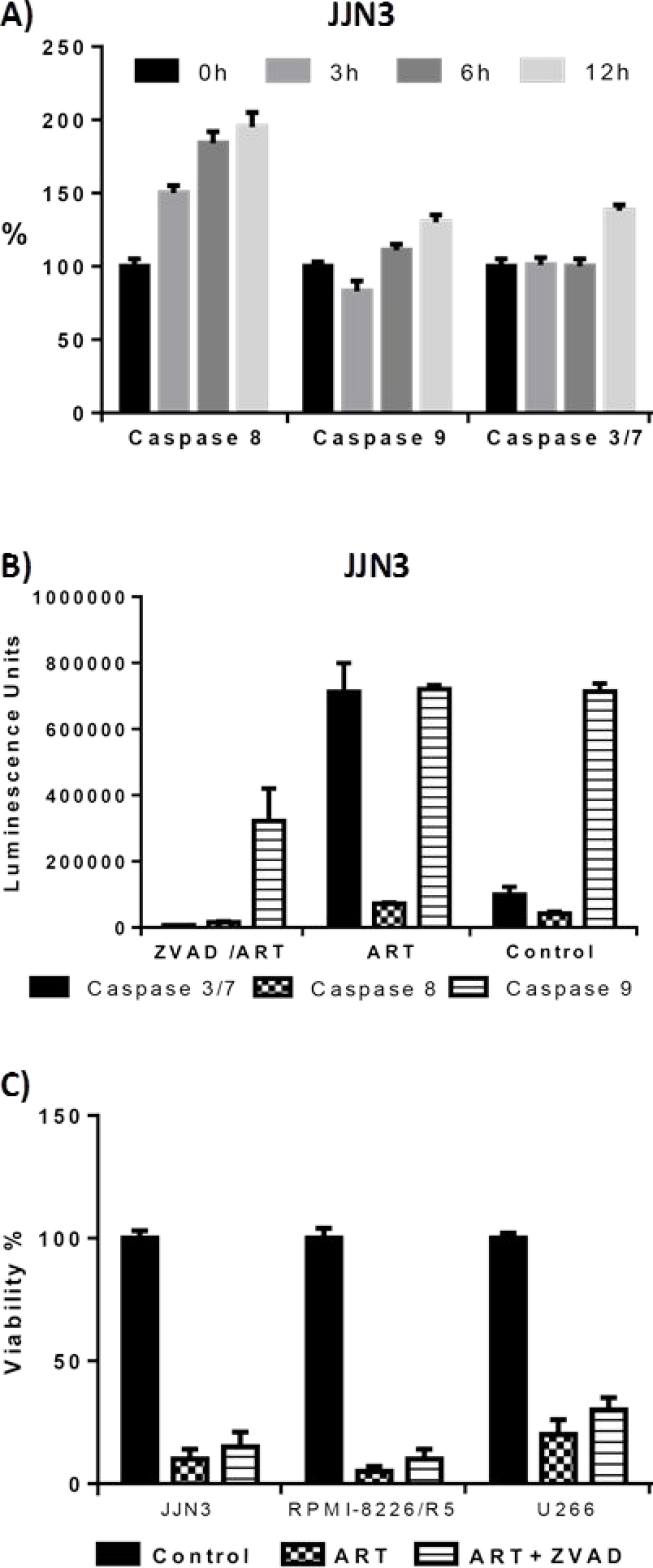
Quantification of activated caspases 3/7, 8, 9 through Caspase-Glo® assay (Figures A-B) and effect of caspase inhibition to ART's effect on cell viability (Figure C) A) Activated caspase 8,9, 3/7 levels compared with untreated cells (0 hour) after 3,6 and 12 hour exposure to 125 μM ART (JJN3 cells) Luminescence was normalized to that of untreated cells. B) Activated caspase levels (units are arbitrary luminescence units) after 48 hour exposure to 125μM ART and 200μM of the pan-caspase inhibitor Z-VADfmk. C) Effect of caspase inhibition to viability of the cells after 48 hour 125μM ART exposure. The effect of ART on cell viability is not affected by inhibition of caspases by Z-VADfmk.

We investigated the mechanism behind ART's ability to induce cell death in MM cells. In several cell lines, flow cytometry analyses of annexin V and PI positivity indicated that ART induced early and late apoptosis (Fig. 2A-E); BZ resistance had no effect on ART's ability to induce apoptosis. To further elucidate ART's mode of action, we determined the sequence and level of caspase activation. ART exposure initiated the extrinsic pathway of apoptosis with a brisk increase in levels of activated caspase-8 at 3 h, followed by activation of caspase-9 at 6 h (Fig. 3A). A prominent increase in the levels of effector caspase 3/7 was evident after 12 h of ART exposure (Fig. 3A). To further examine the role of caspases in inducing apoptosis after ART exposure, we evaluated the impact of pan-caspase inhibitor Z-VAD-mfk on the effectiveness of ART. Z-VAD-mfk effectively inhibited caspase activation by ART (Fig. 3B) but did not block ART's effects on cell viability—at least 70% of ART's effect was retained in JJN3, U266, and RPMI 8226/R5 cells (Fig. 3C). Similar results were obtained in flow cytometry-based apoptosis assays (Supplementary Fig. S2A-B)

### ART-induced non-caspase mediated apoptosis is characterized by cytoplasmic and subsequent nuclear translocation of AIF and EndoG

**Figure 4:**
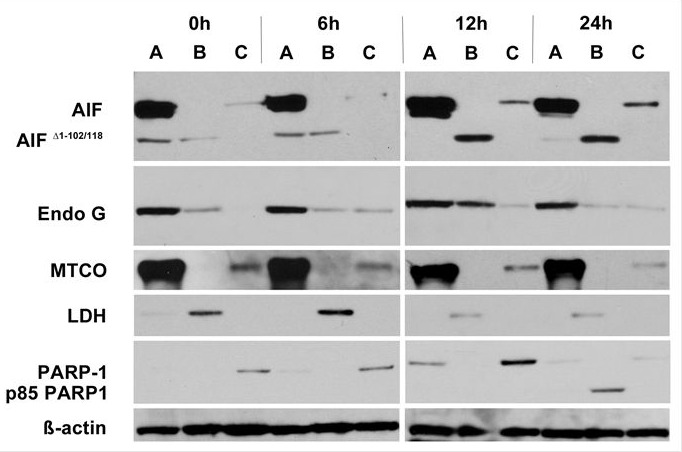
Treatment of JJN3 cells with ART 125μM induces cytoplasmic and nuclear translocation of AIF and EndoG Translocation of AIF and EndoG from the mitochondria to the cytoplasm is detected by Western Blot as early as 6h after exposure to ART. MTCO is used as a mitochondrial marker, LDH is used as a cytoplasmic marker, PARP-1 is used as a nuclear marker and an indicator of apoptosis (cleavage from nucleus and presence of the p85 cleaved form of PARP-1). A: Mitochondrial fraction, B: Cytoplasmic fraction, C: Nuclear fraction

To investigate the molecular effectors involved in ART's induction of non-caspase–mediated apoptosis, we examined the translocation of the two main mitochondrial factors implicated in this type of apoptosis, AIF and EndoG. When JJN3 cells were exposed to 125 μM ART, AIF translocated from mitochondria to cytoplasm at 6 h and to the nucleus at 12 h (Fig. 4); the soluble apoptogenic AIF Δ 1-102/118 isoform was readily detected in the cytoplasmic fraction [12] (Fig. 4). Similarly, EndoG cytoplasmic and nuclear translocation were evident at 6 h and became more prominent at 12 h, especially in the cytoplasmic fraction (Fig. 4). Because nuclear translocation of AIF and EndoG has been directly related to DNA damage and fragmentation [13], we evaluated the cleavage of PARP-1, a nuclear protein that repairs DNA damage [14] and whose cleavage is associated with apoptosis characterized by excessive DNA damage [15]. Indeed, cleavage of PARP-1 in the nuclear fraction was clearly evident at 24 h, well after nuclear translocation of AIF and EndoG. Similar results were obtained for RPMI8226/R5 cells (Supplementary Fig. S3A and B).

### ART primarily targets mitochondria and induces abnormally low levels of ROS in MM cells

**Figure 5:**
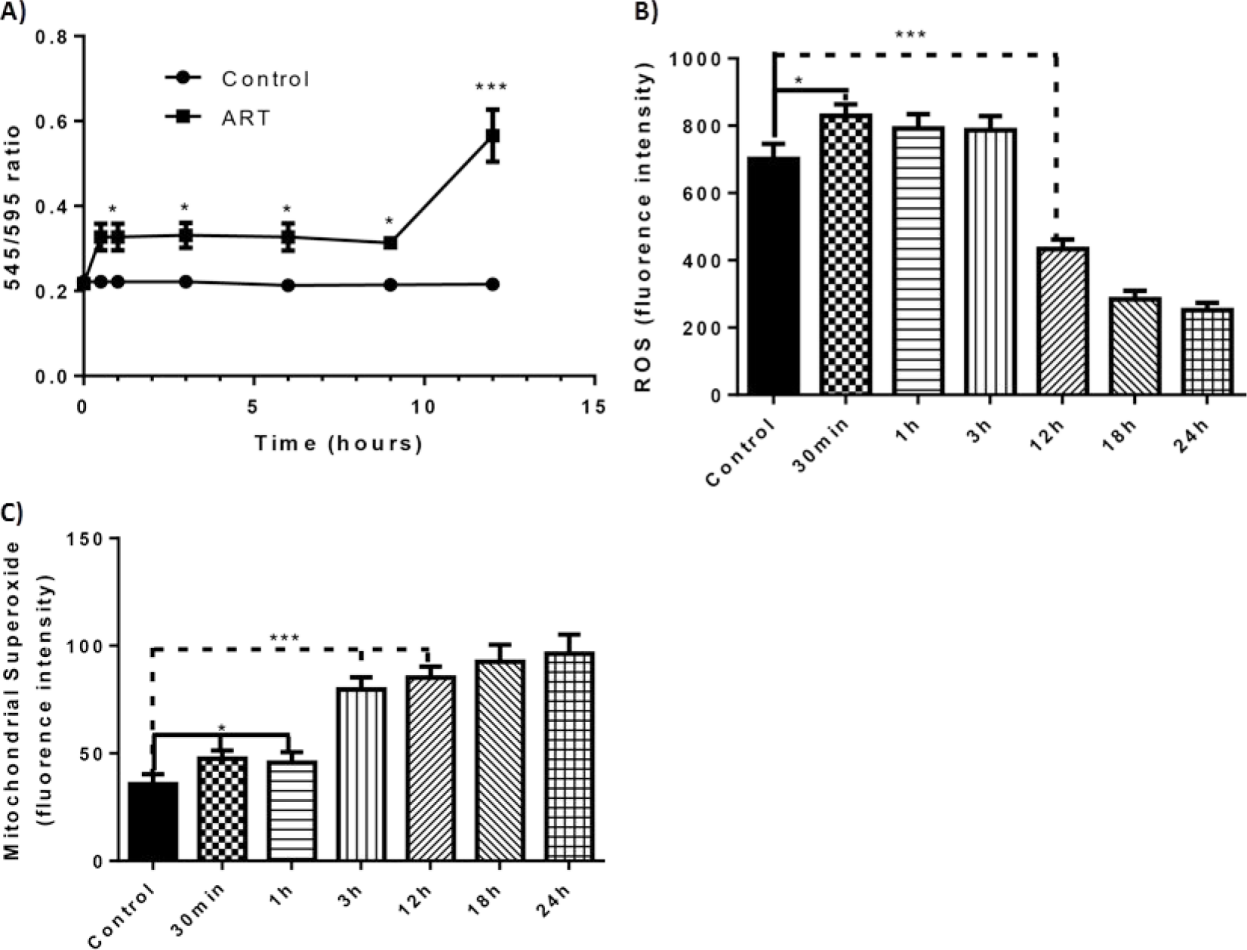
Effect of ART to the mitochondrial membrane potential, cellular level of ROS and mitochondrial superoxide through the course of time and timing of apoptosis A) Change of the mitochondrial membrane potential (ΔΨm) as portrayed through the change of the 545 nm/595 nm ratio in relation to time (JJN3 cells). B) ROS levels (units are arbitrary fluorescence units) at different time intervals of ART 125μM exposure (JJN3 cells) C) Mitochondrial derived superoxide levels (units are arbitrary fluorescence units) at different time intervals of ART 125μM exposure. *P < 0.05 and *P* > 0.01, *** *P* < 0.001

Because ART-mediated apoptosis occurs primarily through the non-caspase pathway, which evolves from mitochondrial factors, we examined the effects of ART on mitochondria by using JC-1 dye [16] to measure changes in mitochondrial membrane potential (ΔΨm) in JJN3 and RPMI-8226 cells. The ratio of the 545-nm (green fluorescence) and 595-nm (red fluorescence) emissions readings was used as a surrogate for the ΔΨm, since this ratio is not affected by factors such as mitochondrial size, shape, and density, which may influence single-component fluorescence signals. As early as 30 minutes after exposure to ART, a small but statistically significant change occurred in the ΔΨm. This change persisted for 12 hours, when a brisk and continuous increase in the ΔΨm was observed (Fig. 5A; Supplementary Fig. S4A).

Because mitochondrial function plays a pivotal role in the cellular ROS balance [17], we examined the effects of ART treatment on the ROS status and production of mitochondria-derived superoxide. ART treatment resulted in a small but steep increase in ROS level within 30 minutes after drug exposure; however, by 12 h, the ROS level was well below that of untreated control cells (Fig. 5B). The decrease in ROS level cannot be due to a reduced number of viable cells because annexin V staining (indicating apoptotic cells) was negligible at the 12-h time point (Supplementary Fig. S4B). Interestingly, the reduced cellular ROS level was accompanied by high levels of mitochondria-derived superoxide (Fig. 5C). The paradoxical reduction in cellular ROS levels and increase in mitochondrial superoxide production that occurred 12 h after ART treatment also occurred in RPMI-8226/R5 MM cells (Supplementary Fig. S4D-E). In contrast, 12 h after treating cells with BZ at a comparable effective dosage (10nM) the ROS level was clearly higher than that of control cells (Supplementary Fig. S4C).

### ART's efficacy depends on the levels of intracellular bivalent iron

An iron dependent mechanism that directly affects the redox cycle of the parasite has been proposed for the antimalarial action of ART [18]. We therefore proceeded to examine the effect of iron in ART's anti-MM efficacy. Supplementation of growth medium with bivalent iron in the form of Iron (II) sulfate heptahydrate at a concentration of 0.8 mg/L (i.e. within the normal range of iron levels in human blood (0.6–1.7 mg/L)) drastically increased ART's anti-MM efficacy, as evidenced by a marked decrease in its IC50 in JJN3, INA-6, RPMI-8286, and U266 cell lines (Supplemental Table S2). This decrease in IC50 was much less pronounced when the protein-bound form of iron, holotransferrin, was used and did not achieve statistical significance when the trivalent form of iron (Iron(III) citrate) was used at the same concentration, as was the bivalent form (Supplemental Table S2). Deferoxamine, an iron chelator that can enter the cells [19], exerted clear antagonism to ART (Supplementary Fig. S5A). The potentiating effect of bivalent iron and the inhibitory effects of deferoxamine were also reflected in levels of ROS and mitochondrial superoxide at the various time points (Supplementary Fig. S5B and C). To clarify whether ART's anti-MM efficacy is potentiated by intracellular or extracellular bivalent iron, we examined the combined effects of ART and EDTA, a potent metal chelator that does not enter the cells [20]. Addition of EDTA had no statistically significant effect on ART's IC50 (Supplementary Fig. S5D), suggesting that it is the intracellular bivalent form of iron which potentiates ART's efficacy.

### ART exhibits synergy with various anti-MM agents

**Figure 6:**
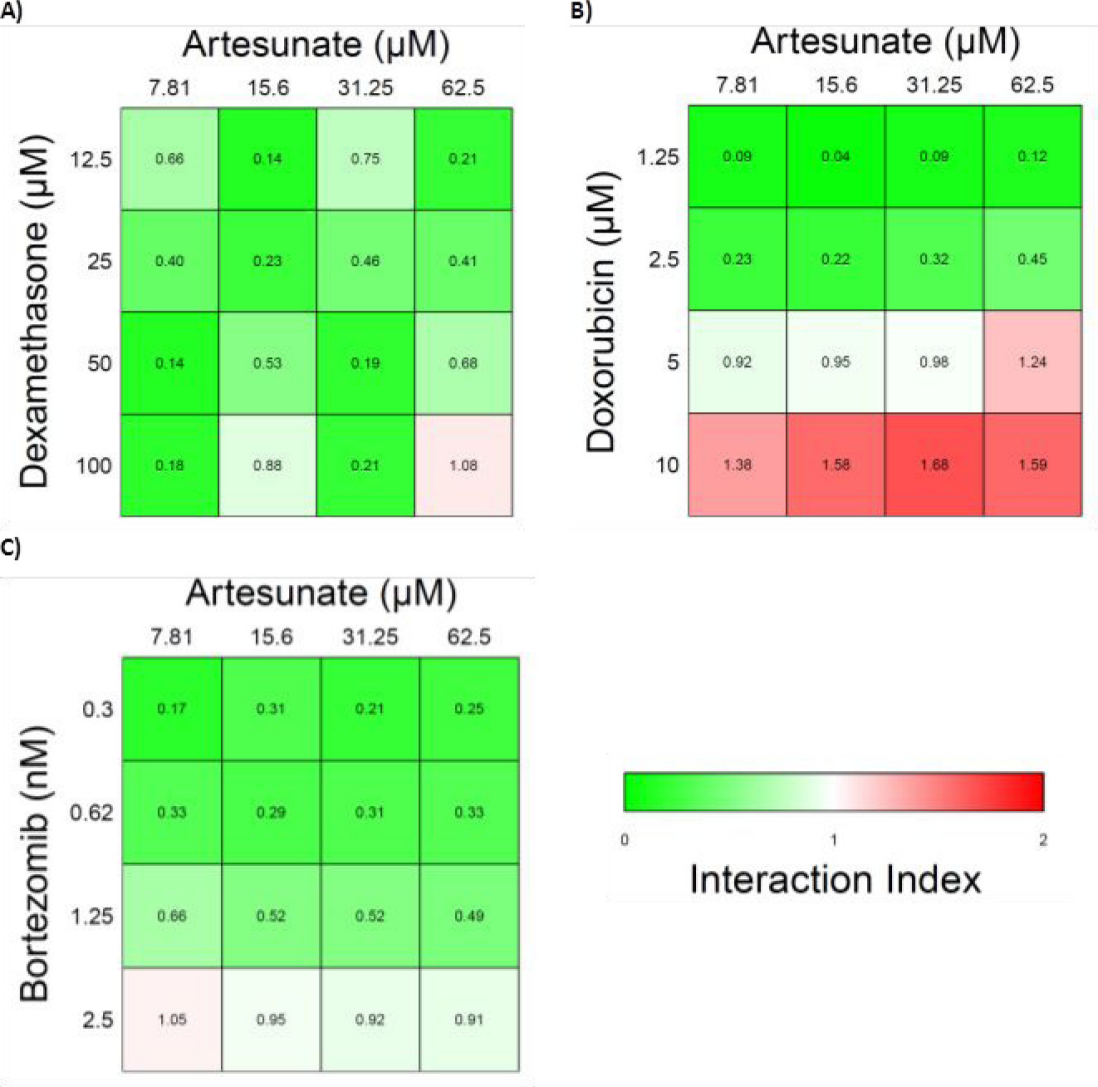
Heatmaps of the interaction index of Artesunate with various frontline anti-MM agents A) Artesunate and Dexamethasone, B) Artesunate and Doxorubicin, C) Artesunate and Bortezomib. Values above 1 (red) are consistent with an antagonistic effect, values equal to 1 (white) are consistent with additive effect and values less than 1 (green) are consistent with a synergistic effect.

Due to the different pathway of inducing apoptosis in ART we then tried to examine the effect of the combination of ART with the known anti-MM agents, DEX, doxorubicin(DOX) and BZ that are known to induce apoptosis through the caspase dependent pathway with a high ROS accumulation. While ART induces apoptosis primarily through a non-caspase dependent pathway, anti-MM agents DEX, doxorubicin(DOX), and BZ induce apoptosis through the caspase dependent pathway characterized by high levels of ROS [21-23]. For this purpose the “Synergy” program was applied as described in the methods section and the Interaction Index (II) was used as an index of interaction evaluation. Values greater than 1 are consistent with antagonistic effect, values equal to 1 are consistent with additive effect and values less than 1 are consistent with a synergistic effect. ART exhibited profound synergism with all tested agents in medium to low dosages. At high dosages the effect was either additive (DEX, BZ) or slightly antagonistic (DOX) (Figure 6A-C). However it should be noted that in the high concentrations the IC50 values were exceeded for all agents.

## DISCUSSION

Artemisinin and its derivative ART have been reported to have anticancer activity in many different in vitro tumor models [6]. This report establishes ART's in vitro effectiveness against MM cells and elucidates the mechanisms by which this agent induces apoptosis of MM cells. Our observations suggest that ART induces apoptosis primarily through the non-caspase mediated pathway and that the mitochondria are ART's primary target in MM cells. Furthermore, initiation of apoptosis was characterized by low levels of cellular ROS levels and a marked increase in mitochondrial superoxide, an effect that has not been previously reported for other drugs.

Non-caspase mediated apoptosis has not been studied extensively [24]. The main factors implicated in this form of apoptosis are the AIF and EndoG [25]. In our experiments ART induced non-caspase apoptosis was related to translocation of AIF and EndoG initially from the mitochondria to the cytoplasm and subsequently to the nucleus. While whether or not non-caspase mediated apoptosis is the dominant pathway of ART induced apoptosis seems to be cell type dependent [7], Zhou et al [26] also found non-caspase apoptosis in lung adenocarcinoma cells as the main mechanism of ART induced cell death. Furthermore, ART caused the release of the apoptogenic isoform Δ 1-102/118 of AIF from the mitochondria, which has been shown to be an aftermath of the outer mitochondrial membrane permeabilization (OMMP) [12]. The OMMP leads to a loss to the ΔΨm and an increase in ROS [27]. It should be noted that these two events coincided early in the timeline of ART exposure and were concurrent with the release of both Δ 1-102/118 AIF and EndoG to the cytoplasm. The nuclear translocation of AIF and EndoG initiated apoptosis in MM cells a finding consistent with the effects of these two factors in other type of cells [28, 29]. Moreover this nuclear translocation preceded the cleavage of PARP-1, in accordance with other reports [30], providing further evidence that the cell death of MM cells from ART exposure is through programmed apoptosis. PARP-1 cleavage, an indicator of late apoptosis [31], followed the nuclear translocation of AIF and EndoG supporting thus the role of these two factors in the ART induced apoptosis in MM cells.

While the biggest difference in ΔΨm was found at 12 hours, changes that were statistically significant were found as early as 30 minutes after drug exposure. This was accompanied by a small but brisk increase in the ROS level of the cells. It should be noted that while the mitochondria are the primary target of ART, for the caspase dependent pathway of apoptosis it is the extrinsic pathway that is activated first. This activation is an early event and occurs at the time of increased ROS production. It has been shown that increased ROS levels can activate the extrinsic pathway of caspase dependent apoptosis by ROS induced sphingomyelinase activation, ceramide production and death receptor clustering [32]. However, at least in MM cell lines, it does not seem to be the increased level of ROS that actually induces apoptosis but the way ART interferes with the redox status of the cell.

The increased mitochondrial superoxide production from the beginning of ART exposure does not translate to an overall increase in cellular ROS levels. In fact the opposite happens; at 12 hours when the mitochondrial derived superoxide level is at its highest, cellular ROS level is well below that of the untreated control. This paradox could imply that ART directly interferes with the Fenton reaction for production of free radicals from superoxide. In this reaction, bivalent iron plays a fundamental role, and inhibition of iron reduction from the bivalent to the trivalent form diminishes production of hydroxyl and free oxygen radicals [33]. This proposed mechanism is supported by our finding that ART's effects on mitochondria and induction of apoptosis are strongly related to intracellular levels of bivalent iron. The interaction of ART with bivalent iron in malarial parasites also has been implicated in the drug's anti-plasmodial effect [34].

Early and sustained mitochondrial superoxide overproduction combined with the early change in the ΔΨm, as observed in our experiments, is consistent with a complex I mitochondrial dysfunction [17]. Interestingly, complex I function is highly dependent on the FMN cofactor, which passes electrons through a chain of seven FeS (iron sulfur) centers to the CoQ reduction site [35], where the bivalent or trivalent state of iron is of crucial importance for the electron transport. Non-caspase mediated apoptosis has been linked with loss of cellular respiration and dysfunction of mitochondrial complex I that precedes nuclear translocation of AIF and EndoG [36]; this is consistent with our findings from MM cell lines treated with ART. In addition, it has been shown that, even in caspase-dependent ΔΨm dissociation, the iron-containing NADH-ubiquinone oxireductase subunit of complex I is primarily affected [37]. Dysfunction of Complex I has been linked with superoxide overproduction along with ΔΨm alterations [38]. Similar findings pertain to the iron-containing subunits of mitochondrial complexes II [39] and III [40]. Also of importance is the fact that heme seems to be one of ART's target molecules in cancer cells, where it reduces heme (bivalent iron) to hemin (trivalent iron) [41]. Heme is essential to many protein complexes and one of them, cytochrome C, plays a fundamental role in the electron transport chain of the mitochondria [42]. Thus the mitochondrion, with its abundance of iron provides an ideal environment for the action of ART. Although we have not shown depletion of bivalent iron as a direct effect of ART, circumstantial evidence - as laid out in this study - suggest Fe^+2^ as a target of ART. We have summarized this in Supplementary Fig. S6. In clinical studies for malaria ART was shown to have an excellent toxicity profile [9, 43], with the most significant finding a transient minimal reduction of the reticulocyte count in a phase I dose escalation study [10]. In vitro studies with human peripheral blood mononuclear cells found the IC50 to be more than 100μM a dosage which as shown here exceeds several times the IC50 in MM cells [44]. When dihydroartemisinin (DHA) - the primary metabolite of ART- was tested in cultures of human CD34+ selected cells only a transient effect on erythroid maturation was evident. No differences in cell number between cells cultured with and without DHA were seen in long term cultures, regardless of the time exposure to DHA [45]. ART was able to overcome resistance in both BZ as well as DEX resistant MM cell lines regardless of whether the resistance was native or acquired. Additionally, we were able to show synergy between ART and several frontline anti-MM agents, which are known to cause caspase-dependent, high-ROS apoptosis. Through these unique mechanisms ART not only has the ability to act independently but can also complement and augment the effect of other anti-myeloma agents. Primary BZ resistance of a subpopulation of MM cells in the clinical setting has been identified as the main mechanism failure of BZ to eradicate the disease [46]. These data provide the rationale for further “*in vivo*” experiments to establish the therapeutic potential of ART either alone or in combination with other agents especially in the relapsed/refractory MM setting.

In conclusion ART is an effective single agent, has synergy with known myeloma agents and can overcome resistance to BZ in preclinical models of MM. One possible explanation for these beneficial effects is that ART induces apoptosis through a unique mechanism of non-caspase mediated apoptosis and low levels of cellular ROS. While more work needs to be done to elucidate the entire mechanism of the ART-induced initial ROS production, mitochondrial dysfunction and the specific redox signaling sequelae that result in apoptosis, and for the establishment of ART's efficacy in MM in vivo, ART could potentially be the forerunner of a whole new class of agents for the treatment of relapsed/refractory MM.

## MATERIALS AND METHODS

### Cell models, cell culture, and experimental conditions

MM cell lines RPMI 8226/R5, INA-6, MM-1S, MM-1R, KMS-11, ARH-77, JJN3, U266; BZ-resistant (BR) sublines JJN3BR and U266BR; and patient-derived primary MM cells were cultured in RPMI-1640 (Corning) supplemented with 10% heat-inactivated fetal bovine serum (Atlas Biologicals), 100 U/ml penicillin/streptomycin, and 2-mM L-glutamine and were incubated at 37°C with 5% CO_2_. Interleukin (IL)-6-dependent cells and purified plasma cells from patients were supplemented with 2-ng/ml IL-6 (R&D Systems). All cell lines were authenticated by a method of copy number variant (CNV) fingerprinting developed by Jonathan Keats and P. Leif Bergsagel [47].

Patient samples were collected under a protocol approved by the Institutional Review Board of the University of Arkansas for Medical Sciences (UAMS). Informed consent was obtained in accordance with the Declaration of Helsinki. Mononuclear cells from bone marrow aspirates were isolated by density gradient centrifugation over Ficoll-Hipaque Plus. Plasma cells were then isolated with immunomagnetic bead selection for CD138+ cells in a Midi MACS LS column following the manufacturer's recommendations (Miltenyi Biotec). Plasma cell purity was confirmed by flow cytometric analysis with phycoreythrin-conjugated CD38 and CD45 antibodies (Miltenyi Biotec).

Stock solutions of ART (Sigma-Aldrich) and BZ (LC Laboratories) were prepared in dimethyl sulfoxide (DMSO) and used as indicated, with a final vehicle concentration that did not exceed 0.5% (vol/vol). All chemicals, unless otherwise indicated, were purchased from Fisher Scientific.

### Cell extract preparation and Western blot analysis

Mitochondrial, cytoplasmic, and nuclear fractions from cultured cell lines JJN3 and RPMI-8226/R5 were isolated with Cell Fractionation Kit Standard from (Mito sciences). Protein samples were fractionated with SDS PAGE and transferred onto PVDF membrane. Antibodies for apoptosis inducing factor (AIF), poly (ADP ribose) polymerase-1 (PARP-1), lactate dehydrogenase (LDH), and mitochondrial cytochrome C oxidase (MTCO) were purchased from Abcam, antibody for β-actin was purchased from Cell Signalling Technology, and the antibody for endonuclease G (EndoG) was purchased from EMD Millipore.

### Cell viability assays

Cell viability was measured with CellTiter 96 AQueous Non-Radioactive Cell Proliferation Assay (MTS) (Promega) as published elsewhere [48]. Briefly, myeloma cells were seeded in 96 wells at a concentration of approximately 2 × 10^4^ cells per well. After incubating in culture medium alone for 24 hours, anti-myeloma agents were added in, in serial 2-fold dilutions titrated according to the potency of each agent. MTS was added to each well at the time points indicated for each experiment. Absorbance was read at a 490 nm wavelength according to the manufacturers recommendations. All tests were done in triplicates.

### Statistical analysis

IC50 calculations and the respective curves were generated by GraphPad Prism v6.0 Software. The effect of the interaction of different agents on combination treatment (synergistic, additive, antagonistic) was analyzed with the freeware program “Synergy” based on the R statistical language (R v2.13.1) as published elsewhere [49]. For each interaction-effect experiment, the Chou-Talalay method [50] was primarily used and results were verified with the Plummer method [51]. For comparisons of treatment groups, unpaired *t*-tests (Mann-Whitney), paired *t*-tests, and one-way or two-way ANOVA (where appropriate) were performed. For ANOVA, Bonferroni posthoc analysis was used to compare treatment groups. All analyses were performed with GraphPad Prism v6.0. Differences with a P value ≤ 0.05 were considered significant.

### Apoptosis assays

Staining cells with annexin V and propidium iodide (PI) for the detection of early and late apoptosis, respectively, was performed according to the manufacturer's specifications (Beckton Dickinson) and as published elsewhere [52]. Data were collected using the FACSuite software on a FACSVerse flow cytometer (Beckton Dickinson). A minimum of 10,000 events were analyzed for each experiment. All experiments were conducted in triplicate.

### Mitochondrial membrane potential (ΔΨm)

The change in the ΔΨm was detected by staining with JC-1 dye (R&D Systems) as described elsewhere [53]. Briefly, after exposure to the indicated drug for the indicated time period, cells were incubated with 3.5 μM JC-1 for 15 minutes at 37°C. After this incubation period cells were then rinsed and transferred to a 96-well plate for fluorescence measurement by a plate reader with the following settings: excitation 485 nm, emission 545 nm and 595 nm, cutoff 530 nm. The ratio of measurements at 545 nm/595 nm was used as the measurement of the ΔΨm.

### ROS and mitochondria-derived superoxide detection and quantification

For the determination of ROS and mitochondrial derived superoxide generation, cells were treated with drugs at indicated doses and time. On the day of the analysis, 0.5x 10^6^ cells were resuspended in 1 mL pre-warmed PBS and incubated with 10 μM H_2_DCFDA (Life Technologies) or in 0.5 ml DPBS with 5 μM MitoSOX (Life Technologies) for 30 min at 37°C. Then, cells were washed with ice-cold PBS and subjected to flow cytometry using the FACSVerse (Becton Dickinson). Data were analyzed with FC Express software (De Novo).

### Caspase activation and quantification assay

Identification and quantification of the activated caspases 3/7, 8, and 9 was performed through the use of the CaspaseGlo assay (Promega), as published elsewhere [54]. Pancaspase inhibitor Z-VAD-fmk was purchased from R&D and was applied at a concentration of 200μM every 24 hours unless otherwise indicated.

## SUPPLEMENTARY FIGURES AND TABLES




