# Supplementary material for: Artesunate overcomes drug resistance in multiple myeloma by inducing mitochondrial stress and non-caspase apoptosis
Source: Oncotarget. 2014 Mar 24;5(12):4118–28. doi: 10.18632/oncotarget.1847 (PMC4147310; doi:10.18632/oncotarget.1847)
Supplement: Supplementary file 1 [file oncotarget-05-4118-s001.pdf]

Artesunate overcomes drug resistance in multiple myeloma by inducing mitochondrial stress and non-caspase apoptosis – Papanikolaou et al

Supplemental Tables

Supplemental Table 1A: ART IC50 in various MM cell lines

Cell Line	IC50µM (48h)	IC50µM (72h)
JJN3	20.7	16.4
JJN3BR	19.5	15.2
U266	28.2	18.6
U266BR	29.7	19.5
MM1S	42.5	21.5
MM1R	40.7	23.6
KMS-11	30.1	24.3
RPMI-8226	8.7	6.7
INA-6	27	18.9
ARH-77	36.7	32.3

Supplemental Table 1B: ART IC50 in primary MM cells

MM primary cells	IC50µM （48h）
Pt1	65.9
Pt2	56.2
Pt3	45
Pt4	30.4
Pt5	30.4
Pt6	20.1

Supplemental Table 2: ART IC50 (72h) in MM cell line cultures supplemented with different forms of iron

MM cell line	IC50 µM (RPMI-1640)	IC50 µM (RPMI+Fe^{+2})	IC50 µM (RPMI+Fe^{+3})	IC50 µM (RPMI+holotransferin)
JJN3	16.4	2.7***	14.6~	7.9***
INA-6	18.9	4.1***	17.5~	12.8**
RPMI-8226	6.7	2.3***	5.1*	3.9*

Fe^{+2} in the iron sulfate heptahydrate form concentration: 0.8mg/L, Fe^{+3} in the iron citrate form concentration: 0.8mg/L, holotransferin concentration: 1 µM.

***p<0.001, **p<0.01, *p<0.05, ~p<0.1 . Comparisons made with the IC50 (72h) on standard RPMI-1640 growth medium supplemented with 10% FBS and 100 U/ml penicillin/streptomycin, 2 mmol/l L-glutamine.
