# Supplementary material for: Artesunate overcomes drug resistance in multiple myeloma by inducing mitochondrial stress and non-caspase apoptosis
Source: Oncotarget. 2014 Mar 24;5(12):4118–28. doi: 10.18632/oncotarget.1847 (PMC4147310; doi:10.18632/oncotarget.1847)
Supplement: Supplementary file 2 [file oncotarget-05-4118-s002.pdf]

Artesunate overcomes drug resistance in multiple myeloma by inducing mitochondrial stress and non-caspase apoptosis – Papanikolaou et al

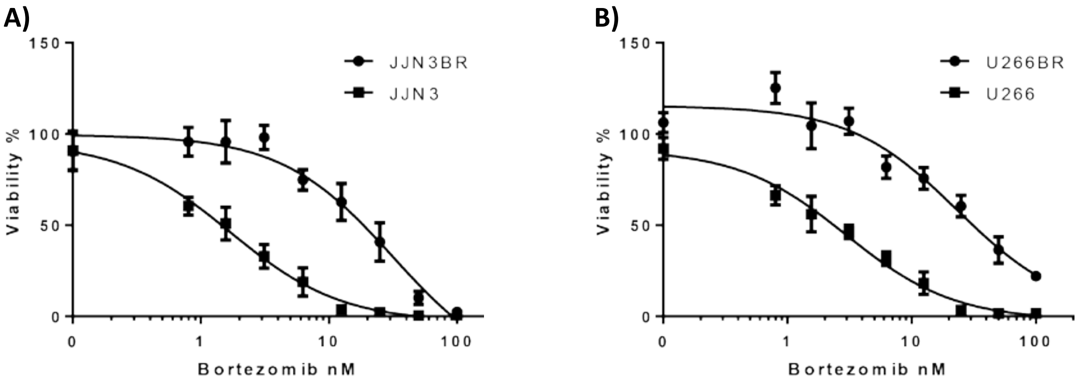

Supplemental Figure 1: IC50 for 48h Bortezomib exposure for the JJN3 (A) and U266 (B) MM cell lines compared to their respective bortezomib resistant sublines depicted in the graph above. IC50 (48h) for JJN3 1.96 nM, IC50 (48h) for JJN3BR 31.77 nM ($p<0.001$). IC50 (48h) for U266 4.75nM, IC50 (72h) for U266BR 47.39 nM ($p<0.001$).

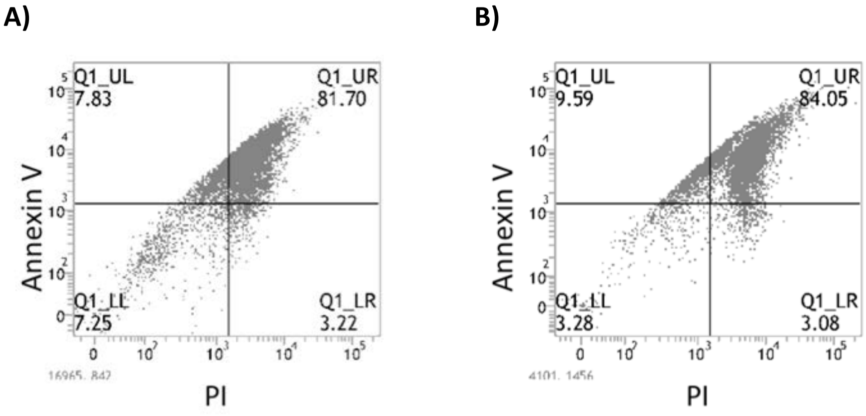

Supplemental Figure 2: Flow cytometry assay for detection of early and late apoptosis A) JJN3 cells after 48 hour exposure to ART 125µM ART and 200µM Z-VADfmk B) RPMI-8226 cells after 48 hour exposure to ART 125µM ART and 200µM Z-VADfmk.

A

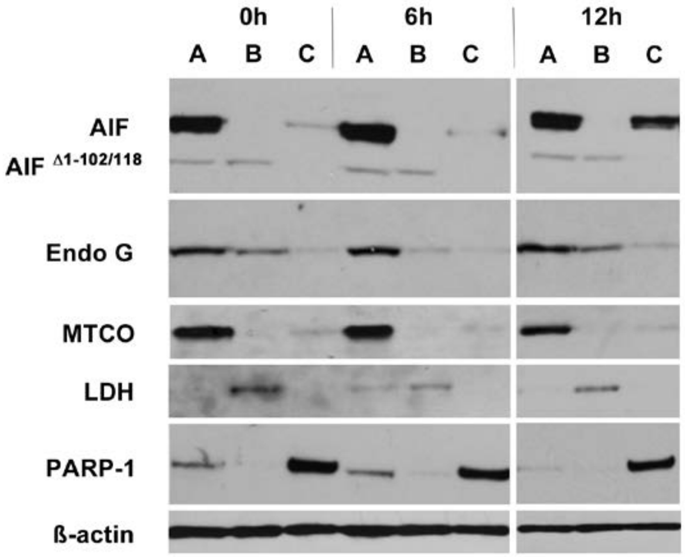

B

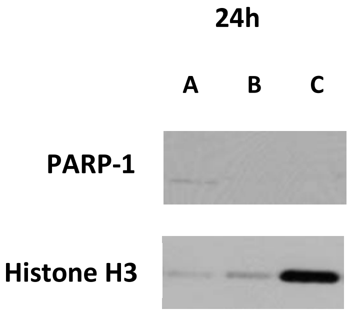

Supplemental Figure 3: Western Blot images of AIF and EndoG cytoplasmic and nuclear translocation after treatment with ART 125µM on RPMI8226/R5 cells. MTCO is used as a mitochondrial marker, LDH is used as a cytoplasmic marker, PARP-1 is used as a nuclear marker and an indicator of apoptosis (cleavage from nucleus). At the 24h exposure to 125µM of ART where the PARP-1 is totally cleaved from the nucleus the Histone H3 is used as a nuclear marker instead. **A:** Mitochondrial fraction, **B:** Cytoplasmic fraction, **C:** Nuclear fraction

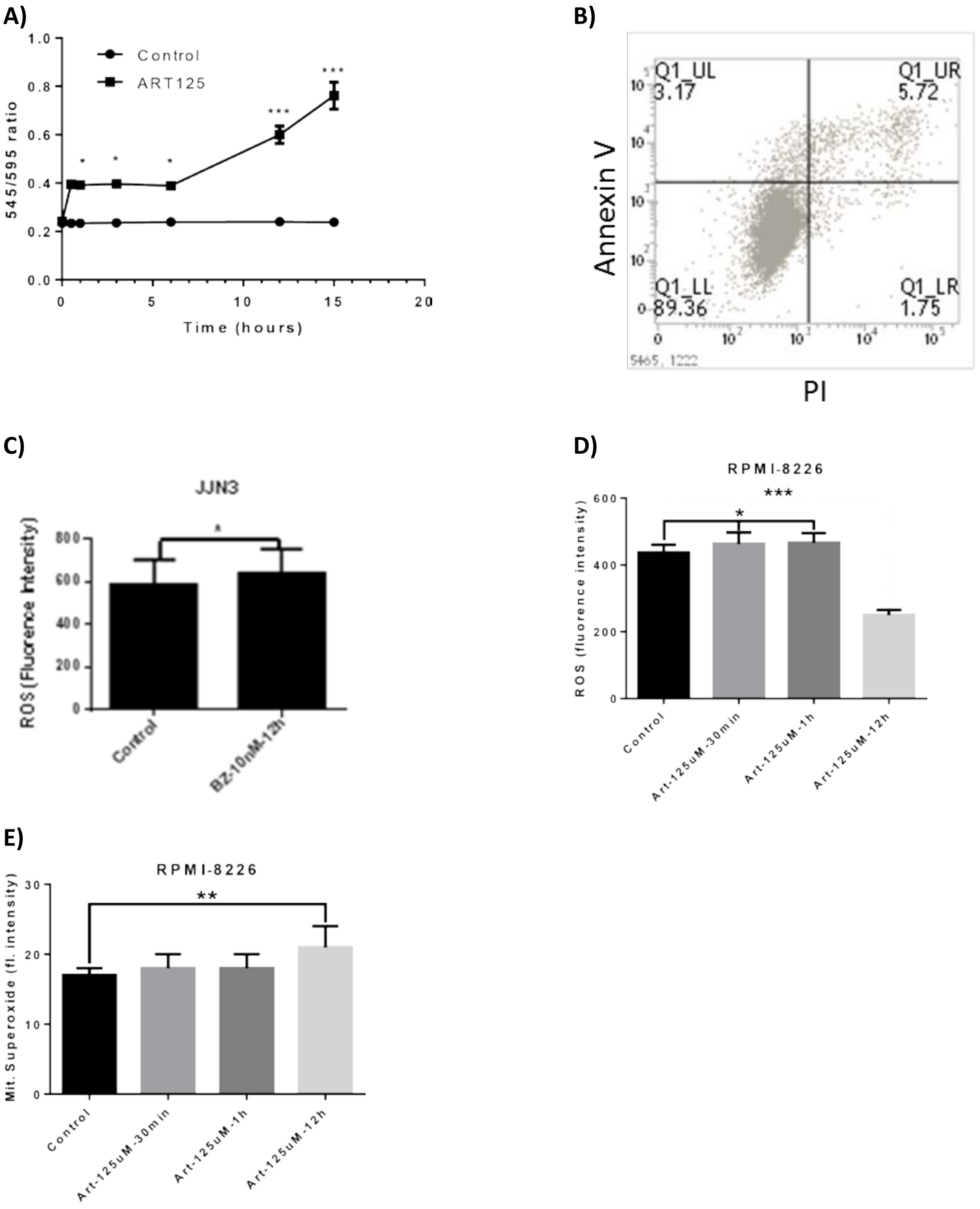

Supplemental Figure 4: A) Change of the mitochondrial membrane potential (ΔΨm) as portrayed through the change of the 545/595 ratio in relation to time (RPMI-8226 cells).B) Annexin V and PI positivity at 12 hours after 125µM ART exposure (JJN3cells), compare with Fig 2A. C) ROS level at the 12h landmark for bortezomib (10nm) (JJN3 cells). D) ROS level for the 30 minutes, 1 hour and 12 hour time points after treatment with ART 125µM (RPMI-8226 cells). E) Mitochondrial Superoxide levels for

the 30 minutes, 1 hour and 12 hour time points after treatment with ART 125 µM (RPMI-8226 cells). * p<0.05 and p>0.01. ** p<0.01 and p>0.001, *** p<0.001

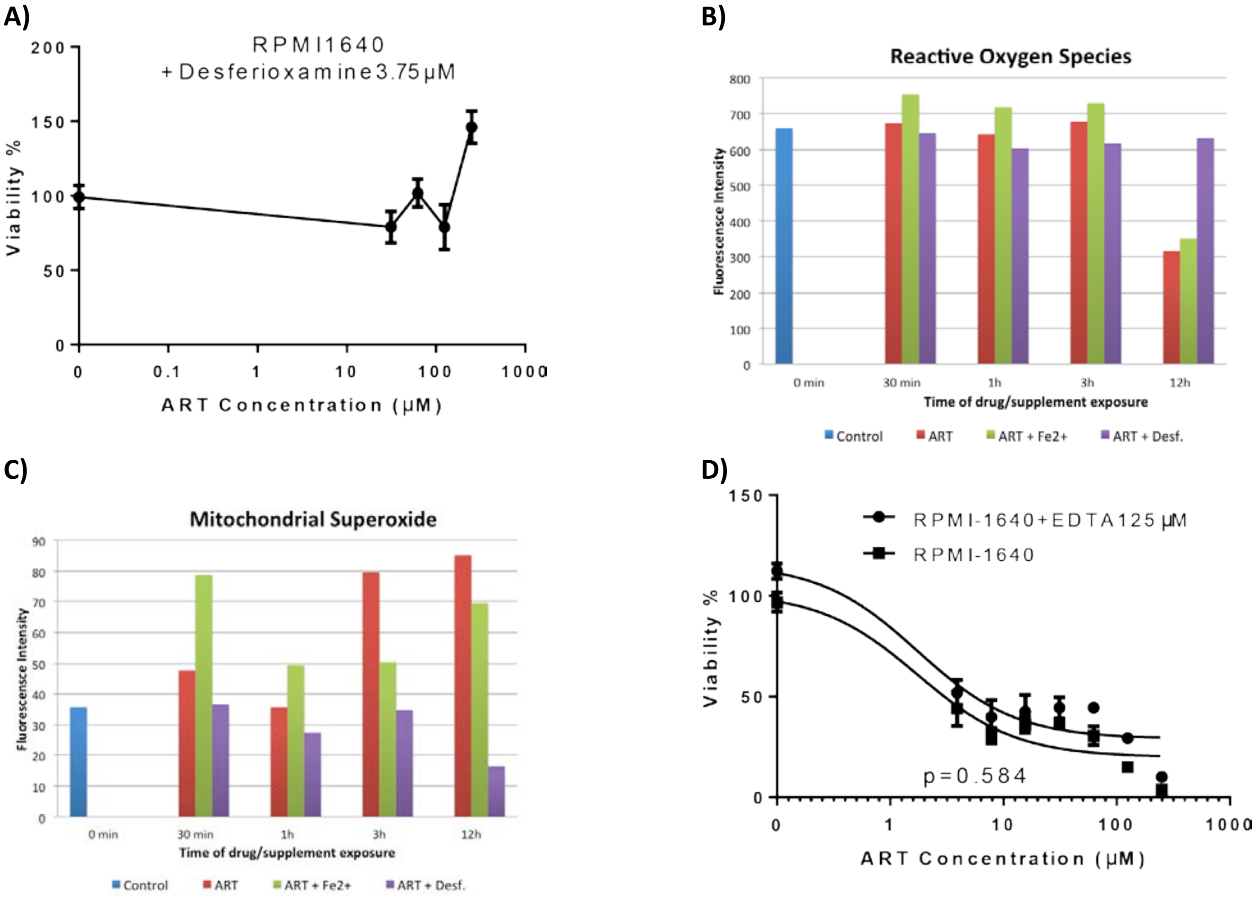

Supplemental Figure 5: A) Viability levels after ART exposure at a concentration of 3.75 µM Desferioxamine (JJN3 cells) B) Effect of bivalent iron (Fe^{+2}, Iron (II) sulfate heptahydrate 0.8 mg/L) and Desferioxamine (Desf, 3.75µM) to the ROS level compared to control (JJN3 cells). (units are arbitrary fluorescence units) C) Effect of bivalent iron (Fe^{+2} , Iron (II) sulfate heptahydrate 0.8 mg/L) and Desferioxamine (Desf, 3.75µM) to the Mitochondrial Superoxide level compared to control (JJN3 cells). (units are arbitrary fluorescence units) D) IC50 curve of ART in the presence of 62.5µM EDTA (JJN3 cells). *p<0.05 and p>0.01, ***p<0.001

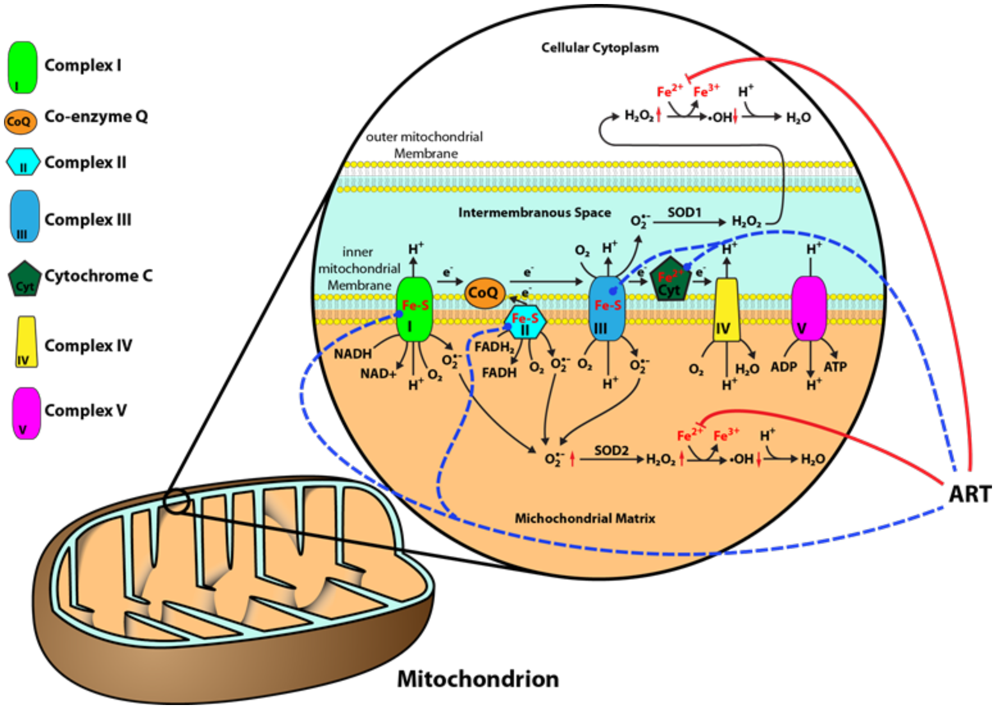

Supplemental Figure 6: Proposed model for the mechanism of action of ART for inducing increased mitochondrial superoxide with low cellular ROS. ART's ability to interfere with bivalent iron induces dysfunction of the mitochondrial respiratory chain particularly the Fe-S containing complexes I, II and III and superoxide overproduction (blue dashed line). The heme containing cytochrome C probably is also affected by reduction of heme to hemin by ART (blue dashed line). Concurrently due to the depletion of bivalent iron, the Fenton reaction of ROS generation from hydrogen superoxide is inhibited (red continuous line), thus leading to the "paradox" of increased mitochondrial superoxide and low cellular ROS.
